# A Potent Histone Deacetylase Inhibitor MPT0E028 Mitigates Emphysema Severity via Components of the Hippo Signaling Pathway in an Emphysematous Mouse Model

**DOI:** 10.3389/fmed.2022.794025

**Published:** 2022-05-18

**Authors:** Lu-Yang Yeh, Yu-Ting Fang, Hong-Sheng Lee, Chia-Hao Liu, You-Yin Chen, Yu-Chun Lo, Vincent Laiman, Jing-Ping Liou, Kian Fan Chung, Hsiao-Chi Chuang, Chien-Huang Lin

**Affiliations:** ^1^School of Medicine, College of Medicine, Taipei Medical University, Taipei, Taiwan; ^2^Department of Biomedical Engineering, National Yang Ming Chiao Tung University, Taipei, Taiwan; ^3^Graduate Institute of Medical Science, College of Medicine, Taipei Medical University, Taipei, Taiwan; ^4^PhD Program for Neural Regenerative Medicine, College of Medical Science and Technology, Taipei Medical University, Taipei, Taiwan; ^5^International PhD Program in Medicine, College of Medicine, Taipei Medical University, Taipei, Taiwan; ^6^Department of Anatomical Pathology, Faculty of Medicine, Public Health, and Nursing, Universitas Gadjah Mada, Yogyakarta, Indonesia; ^7^School of Pharmacy, College of Pharmacy, Taipei Medical University, Taipei, Taiwan; ^8^National Heart and Lung Institute, Imperial College London, London, United Kingdom; ^9^School of Respiratory Therapy, College of Medicine, Taipei Medical University, Taipei, Taiwan; ^10^Division of Pulmonary Medicine, Department of Internal Medicine, Shuang Ho Hospital, Taipei Medical University, New Taipei City, Taiwan; ^11^Cell Physiology and Molecular Image Research Center, Wan Fang Hospital, Taipei Medical University, Taipei, Taiwan

**Keywords:** alveolar, apoptosis, COPD, HDAC, inflammation, TAZ

## Abstract

**Background:**

Chronic obstructive pulmonary disease (COPD) is a major cause of chronic mortality. The objective of this study was to investigate the therapeutic potential of a novel potent histone deacetylase (HDAC) inhibitor MPT0E028 on emphysema.

**Materials and Methods:**

A mouse model of porcine pancreatic elastase (PPE)-induced emphysema was orally administered 0, 25, or 50 mg/kg body weight (BW) of the MPT0E028 five times/week for 3 weeks. Pulmonary function, mean linear intercept (MLI), chest CT, inflammation, yes-associated protein (YAP), transcriptional coactivator with PDZ-binding motif (TAZ), surfactant protein C (SPC), T1-α, p53, and sirtuin 1 (SIRT1) levels were examined.

**Results:**

50 mg/kg BW of the MPT0E028 significantly decreased the tidal volume in emphysematous mice (*p* < 0.05). Emphysema severity was significantly reduced from 26.65% (PPE only) to 13.83% (50 mg/kg BW of the MPT0E028). Total cell counts, neutrophils, lymphocytes, and eosinophils significantly decreased with both 25 and 50 mg/kg BW of the MPT0E028 (*p* < 0.05). Also, 50 mg/kg BW of the MPT0E028 significantly decreased the levels of KC, TNF-α, and IL-6 in lung tissues and serum (*p* < 0.05). Expressions of p-TAZ/TAZ in lung tissues significantly decreased with 50 mg/kg BW of the MPT0E028 (*p* < 0.05). Expressions of p53 significantly decreased in alveolar regions with 50 mg/kg BW of the MPT0E028 (*p* < 0.05), and the expression of SPC increased in alveolar regions with 50 mg/kg BW of the MPT0E028 (*p* < 0.05).

**Conclusions:**

Our study showed that the potent HDAC inhibitor MPT0E028 reduced the severity and inflammation of emphysema with improvement in lung function, which could be regulated by Hippo signaling pathway. The MPT0E028 may have therapeutic potential for emphysema.

## Introduction

Chronic obstructive pulmonary disease (COPD), a mixture of small-airway disease and emphysema, is defined by the presence of a low ratio of the forced expiratory volume in 1 s (FEV1) to the forced vital capacity (FVC) assessed by spirometry (1). COPD is now one of the top three causes of death all over the world, especially in low- and middle-income countries (2). In addition, 10.1% of population in the world has COPD grade 2 or higher with 11.8% for men and 8.5% for women (3), resulting in more than 3 million people dying from it each year. Although COPD is an important clinical and public health issue, there are currently still limited treatment strategies.

COPD is characterized by destruction of the small airways and defective tissue repair in the alveolar compartment, which lead to bronchitis, small-airway remodeling and emphysema. These are crucial problems underlying the pathogenesis of COPD. Sustained inflammation derived from the irritants around type 2 alveolar epithelial cells (AECIIs) and type 1 AECs (AECIs) cause epigenetic changes in the chromatin of immune cells, which then contribute to the dysregulation of inflammatory responses and stimulate the generation of inflammatory chemokines in the human lungs (4). Infiltration of inflammatory cells in the lungs, including neutrophils, macrophages, lymphocytes and mast cells, directly causes structural changes by secreting enzymes and inflammatory cytokines or indirectly by regulating other cellular functions. Consequently, loss of alveolar cell attachment to the small airways and decreased lung elastic recoil are present and affect daily symptoms (5, 6). Moreover, increasing lung eosinophil numbers, which are associated with increased corticosteroid responsiveness (7), are also detected and might be useful when selecting different therapeutic approaches for COPD patients.

The Hippo signaling pathway was reported to be involved in regulation of lung disease (8). It controls development of animal organ, growth, and regeneration upon injury, and it also involved in tumorigenesis in mammals. The transcriptional coactivators, yes-associated protein (YAP) and transcriptional coactivator with a PDZ-binding motif (TAZ) are downstream effectors in this cascade (9). When the Hippo pathway is turned on, the large tumor suppressor (Lats) 1/2 kinase directly phosphorylates and inhibits YAP/TAZ. The deficiency of TAZ showed abnormal alveolarization and caused adult mice airspace enlargement mimicking emphysema (10). On the contrary, when the Hippo pathway is turned off, YAP/TAZ are dephosphorylated, which induces their nuclear accumulation and activates expressions of genes promoting cell proliferation and inhibiting apoptosis (11). Furthermore, YAP and TAZ are required for normal lung development and regeneration (12), including early airway branching morphogenesis, epithelial lineage differentiation, and cellular transition to air breathing (13). As previous studies showed, inactivation of Hippo signaling increased β-catenin and Fgf10 expressions, which indicates the cytoplasmic role of YAP in committing to an epithelial lineage, and promotes the proliferation and differentiation of surfactant protein C (SPC)-expressing AECIIs after infection (14, 15). Furthermore, mice that lacked YAP/TAZ in AECIIs presented prolonged inflammatory responses in lung tissues and delays in repairing the alveolar epithelium after bacterial infection (15). It was also reported that components of the Hippo signaling pathway can lead to failure of alveolar development and abnormalities of lung epithelial cells, that contribute to emphysema or pulmonary cysts (16).

Histone acetyltransferase (HAT) and histone deacetylase (HDAC) are families of nuclear enzymes that modify the transcription of inflammatory genes by regulating the chromatin structure (17). Acetylation and deacetylation of core histones by HAT and HDAC lead to changes in the chromatin structure and affect gene transcription. They contribute to the development of diseases such as cancer and chronic inflammation (i.e., COPD) by altering the affinity of transcription factors and RNA polymerase II to DNA. In individuals with COPD, the reduced activity of HDAC2 (18), which deacetylates histone 4 (H4) at the IL-8 promotor, is correlates with steroid resistance and proposed as biomarker for disease severity (19). However, HDAC3, the other type of HDAC family, regulates interleukin (IL)-1-induced gene expression by removing inhibitory nuclear factor (NF)-κB p65 acetylation at K122, 123, 314, and 315 (20), which is involved in the pathology of COPD. In addition to HDAC3, HDAC6 and HDAC8 are also key roles in regulation of inflammation. HDAC8 is known to promote actin filament polymerization and subsequent smooth muscle contraction, which plays an important role in airway inflammation and remodeling (21), while HDAC6 leads to promote microtubules, and thereby increase cellular motility (22). With HDAC inhibitors, the deacetylation of histone proteins are downregulated to maintain a balance between pro- and anti-inflammatory gene expressions (23). Our previous study showed that a novel potent HDAC inhibitor MPT0E028 was able to suppress different types of cancer (24), and B-cell lymphoma (25). A HDAC inhibitor increased the expression of proSFTPC and secretoglobin family 3A member 2 (SCGB3A2) in diseased adult lung tissues, indicating the restoration of alveolar and airway differentiation and regeneration (26). In animal models of cigarette smoke-induced airway inflammation, attenuated inflammatory gene expression and inflammatory cell recruitment were observed with the selective inhibitor of HDAC1, 2 and 3 (27). This might provide a novel and effective method toward developing treatments for inflammatory lung diseases.

Clinically, beta-2 agonists and antimuscarinic drugs as bronchodilators increase the FEV1, and combinations of long-acting muscarinic antagonists and inhaled corticosteroids to reduce exacerbation are used to control COPD. We have successfully developed the potent HDAC inhibitor MPT0E028 for cancer therapy; however, the effects of the MPT0E028 on chronic lung disease remain unclear. The objective of this study was to investigate the effects of the MPT0E028 on emphysema severity. The roles of components of the Hippo signaling pathway in emphysema were investigated.

## Materials and Methods

### Animals

Seven-week-old C57BL/6JNar male mice were obtained from the National Laboratory Animal Center (Taipei, Taiwan) and housed at the laboratory animal center of Taipei Medical University under conditions of 22 ± 2°C, 55% ± 10% humidity, and a 12-h dark/light cycle. This study was approved by the institutional Animal Care and Use Committee of Taipei Medical University (no. LAC-2019-0256).

### Emphysematous Mouse Model

**Figure 1:**
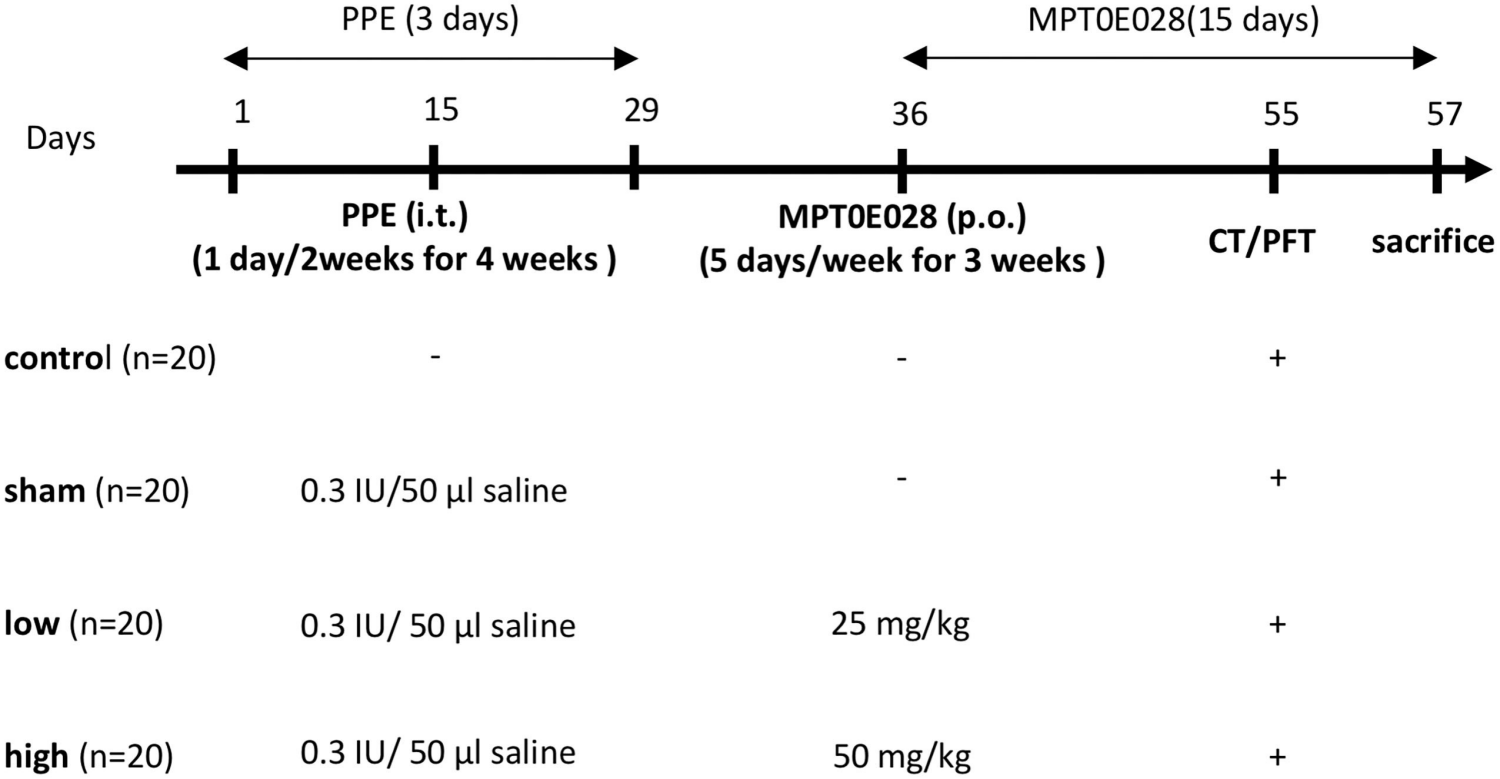
Illustration of the experiment design. Mice in the emphysema group (*n* = 60) were intratracheally instilled with 0.3 IU/50 μl phosphate-buffered saline (PBS) containing porcine pancreatic elastase (PPE) 1 day/2 weeks for 4 weeks. Mice in the control group (*n* = 20) received 50 μl of PBS at the same intervals. After PPE administration, mice in the emphysema group were randomly orally administrated 0 (sham group), 25 (low group), or 50 mg/kg body weight (high group) of the MPT0E028 five times/week for 3 weeks. Lung function tests and chest CT images were conducted a day before euthanasia.

The emphysematous model was conducted as described in a previous report (28). Mice were randomly assigned to a control group (*n* = 20) and an emphysema group (*n* = 60). Mice in the emphysema group received intratracheal (IT) instillation of 0.3 IU/50 μl phosphate-buffered saline (PBS) of porcine pancreatic elastase (PPE; Sigma-Aldrich, St. Louis, MO, USA) by micro-spray three times at 2-week intervals under general anesthesia with 3% isoflurane (Figure 1). Mice in the control group received 50 μl of PBS by IT instillation with the same time intervals. The body weight (BW) was measured before administrating PPE and the MPT0E028, and before the mice were euthanized.

### Experimental Design

The experimental design is shown in Figure 1. Mice in the emphysema group were randomly administered the oral MPT0E028 (98% purity (E)-3-[1-(benzenesulfonyl)-2,3-dihydroindol-5-yl]-N-hydroxyprop-2-enamide (Molecular Formula: C17H16N2O4S); provided by Prof. Ching-Ping Liu, Taipei Medical University, Taipei, Taiwan) (24) at 0 (sham group), 25 (low group), or 50 mg/kg BW (high group) five times/week for 3 weeks according to previous study (24). Facial blood, lung function test, and chest computed tomographic (CT) images were taken a day before euthanasia. The mice were then divided into 2 groups for histology (*n* = 10) prepared in formaldehyde and lung sample collection in −80°C (*n* = 10), respectively. Bronchial alveolar lavage fluid (BALF) was collected immediately after euthanasia for the analysis of the cell counts and supernatant and processed as described previously (29). We also took the entire lungs for histological analysis and to collect lung lysates (30).

### Pulmonary Function Test

Non-invasive pulmonary function was measured with a whole-body plethysmography system (Buxco Electronics, Wilmington, NC, USA) to calculate the change of pressure inside the chamber. A mouse was placed into the system for 5 min, and data of the frequency, tidal volume, expiratory flow at 50% expired volume, and minute ventilation were collected.

### MicroCT Image Acquisition

Mice were anesthetized and placed in a chamber for *in vivo* CT (Skyscan 1176, Kontich, Belgium). According to the manufacturer's instructions, the CT scanner was periodically calibrated. A water-containing Eppendorf tube was used as a phantom to calibrate the image correlating with Hounsfield units (HU). Images were acquired in the list mode with the following parameters: 50 kVp x-ray source voltage, 500 μA current, a composite x-ray filter of 0.5 mm aluminum, 87 ms camera exposure time per projection, projections acquired at 0.7° increments over a total angle of 180°, and images produced with a real pixel size of 34.75 μm. Reconstructed images had a total of 573 slices with an isotropic 34.79 μm voxel size and 864 × 852 image resolution.

### CT Image Quantitative Analysis

Avizo 7.0, for 3D visualization and analysis (FEI Visualization Sciences Group, Burlington, MA, USA) was adopted to perform a quantitative analysis of the extent of emphysema on CT images. First, we used a threshold procedure to extract the entire lung field from CT images. The selected threshold was set to a range of −900 to −200 HU, because intensity values below −900 HU are rare in scans of healthy mice (the volume below −900 HU was <5% of the total lung volume in all healthy animals of any age) (31). Then, the airways were segmented using a region-growing method for airway tree segmentation (32). The algorithm is based on the first candidate region of an airway from CT images by planting a seed and propagating a voxel comparison algorithm that automatically searches the second candidate region. As the seed grows, it connects similar voxels of adjacent regions and obtains a three-dimensional (3D) model of the airway branches. Airway branches were removed from the entire lung volume before further emphysema quantification. Because previous reports revealed that areas with < −600 HU are significantly increased in parallel with the degree of emphysematous lung (33), the final step was to set a low attenuation area (LAA) at −871 to −610 HU as the area of emphysema and to form segments for 3D quantification.

### Hematology

White blood cells, neutrophils, lymphocytes, and eosinophils in the BALF were analyzed with a hematology analyzer (IDEXX Laboratories, Westbrook, ME, USA). Data are presented as differential cell counts.

### Enzyme-Linked Immunosorbent Assay

An ELISA was conducted to determine levels of interleukin (IL)-6 (ThermoFisher Waltham, MA, USA), keratinocyte-derived chemokine (KC) (R&D Systems, Minneapolis, MN, USA), tumor necrosis factor (TNF)-α (ThermoFisher), and matrix metallopeptidase (MMP)-12 (FineTest, Wuhan, China) in BALF, lung lysates, and serum. The analyses were conducted in accordance with the manufacturer's instructions.

### Western Blot Analysis

Protein samples of lung lysates were separated on 10% sodium dodecylsulfate polyacrylamide gel electrophoresis (SDS-PAGE) with the Bio-Rad system followed by transfer to polyvinylidene difluoride (PVDF) membranes (Millipore, Darmstadt, Germany). After blocking the PVDF membranes with 5% milk in TBST, primary antibodies of YAP (1:500), p-YAP (1:1000), T1α (1:1000) (Abcam, Cambridge, UK), TAZ (1:1000), p-TAZ (1:1000), sirtuin 1 (SIRT1) (1:1000), p53 (1:1000) (Cell Signaling Technology, Massachusetts, USA), SPC (1:5000) (SAB, Maryland, USA), and β-actin (1:5000) (GeneTex, California, USA) were used. The membranes were then incubated with anti-rabbit (1:5000) or anti-mouse (1:5000) secondary antibodies, followed by incubation with an enhanced chemiluminescence (ECL) reagent. Images were taken with the BioSpectrum Imaging System (UVP, Upland, CA, USA) and analyzed using Image-Pro vers. 4 (Media Cybernetics, Rockville, MD, USA).

### Immunohistochemical Assays

Expressions of T1α, SPC, p53, and p-YAP in lung tissues embedded in paraffin were analyzed by IHC assays with the Novolink polymer detection system (Leica Biosystems, Buffalo Grove, UK). Briefly, an antigen was retrieved on a deparaffinized and rehydrated lung slice with a citrate buffer solution at 60°C for 10 min. After blocking, primary antibodies were diluted at 1:500 and applied overnight at 4°C. Next, a horseradish peroxidase-conjugated second antibody was added, and visible color was revealed with DAB detection kit (Leica Biosystems, Buffalo Grove, UK). Images were obtained from the MoticEasyScan system (Motic, Kowloon City, Hong Kong). Quantification of protein expressions in the images was conducted with Fiji Sc software (National Institute of Health, Bethesda, MD, USA) to analyze the density of expression of cells in regions of interest (34), and we selected 10 cells for each slide.

### Mean Linear Intercept

Emphysema severity was assessed using the MLI (35). Lung sections embedded in paraffin wax were stained by hematoxylin and eosin (H&E). We evaluated the alveolar size by the equation: Lm = (0.57/average intercept) ×1,000 (μm) as the direct index of the severity of emphysema.

### Statistical Analysis

All data are presented as the mean ± standard deviation (SD). The outliers were identified as < first quartile (Q1)-1.5^*^interquartile range (IQR) and > third quartile (Q3)+1.5^*^IQR using GraphPad Prism 7 (GraphPad Software, San Diego, CA, USA). Multiple variables were compared using a one-way analysis of variance (ANOVA) with Tukey's *post-hoc* test. Data analysis was performed using GraphPad Prism 7. *p* < 0.05 was set as statistical significance.

## Results

### The MPT0E028 Improved the Body Weight

**Figure 2:**
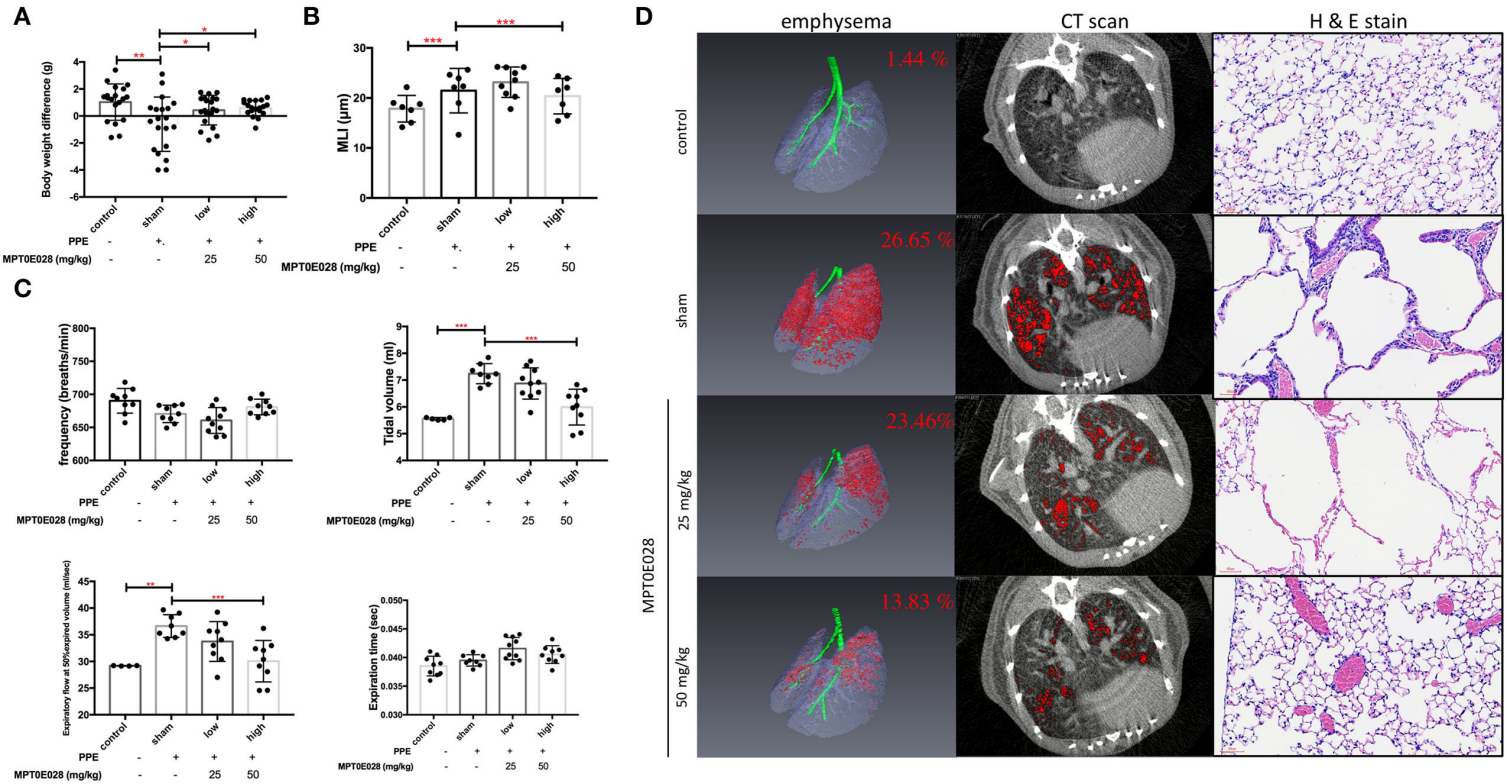
Pulmonary pathophysiology by the MPT0E028 in mice with porcine pancreatic elastase (PPE)-induced emphysema. **(A)** Body weight (BW) differences after treatment by the MPT0E028 at 0, 25, and 50 mg/kg BW (*n* = 20). **(B)** Emphysema score of the mean linear intercept (MLI) by t the MPT0E028 at 0, 25, and 50 mg/kg BW (*n* = 3). **(C)** Non-invasive pulmonary function test after administration of the MPT0E028 at 0, 25, and 50 mg/kg BW (*n* = 10). **(D)** Emphysema severity by chest CT after treatment with the MPT0E028 at 0, 25, and 50 mg/kg BW (*n* = 3). * *p* < 0.05; ** *p* < 0.01; *** *p* < 0.001.

Figure 2A demonstrates changes in BWs in this study. As shown in Supplement Information 1, BWs were significantly reduced in mice with PPE-induced emphysema compared to the control (*p* < 0.01). After 3 weeks of administrating the MPT0E028, we observed that BWs of mice with PPE-induced emphysema were significantly increased by the high-dose treatment (50 mg/kg BW) of the MPT0E028 (*p* < 0.05).

### Effects of the MPT0E028 on Lung Function

Pulmonary functions, including the frequency, tidal volume, expiratory flow at 50% expired volume, and expiration time, were measured as shown in Figure 2C. We observed that the tidal volume and expiratory flow at 50% expired volume had significantly increased in emphysematous mice compared to the control group (*p* < 0.01). Increasing levels of lung functions were significantly reversed by 50 mg/kg BW of the MPT0E028 (*p* < 0.001). There was no significant difference in the frequency or expiration time.

### The MPT0E028 Reduced Emphysema Severity

Figure 2D illustrates emphysema severity in mice. A significant increase in the ratio of emphysema to the effective capacity in emphysematous mice by 25.21% was observed (*p* < 0.001). In addition, we found that the ratio of emphysema to the effective capacity was reduced by 3.19% by 25 mg/kg BW of the MPT0E028, and significantly decreased by 12.82% by 50 mg/kg BW of the MPT0E028 (*p* < 0.01), in comparison with emphysematous mice.

### The MPT0E028 Improved the MLI in Emphysematous Mice

The length of MLI in emphysematous mice is demonstrated in Figure 2B. We discovered that the MLI on H&E-stained slides of mice with PPE-induced emphysema significantly increased compared to the control group (*p* < 0.001). It significantly decreased after 50 mg/kg BW of the MPT0E028 was administered (*p* < 0.001).

### The MPT0E028 Reduced Inflammatory Cell Infiltration Into the Lungs

**Figure 3:**
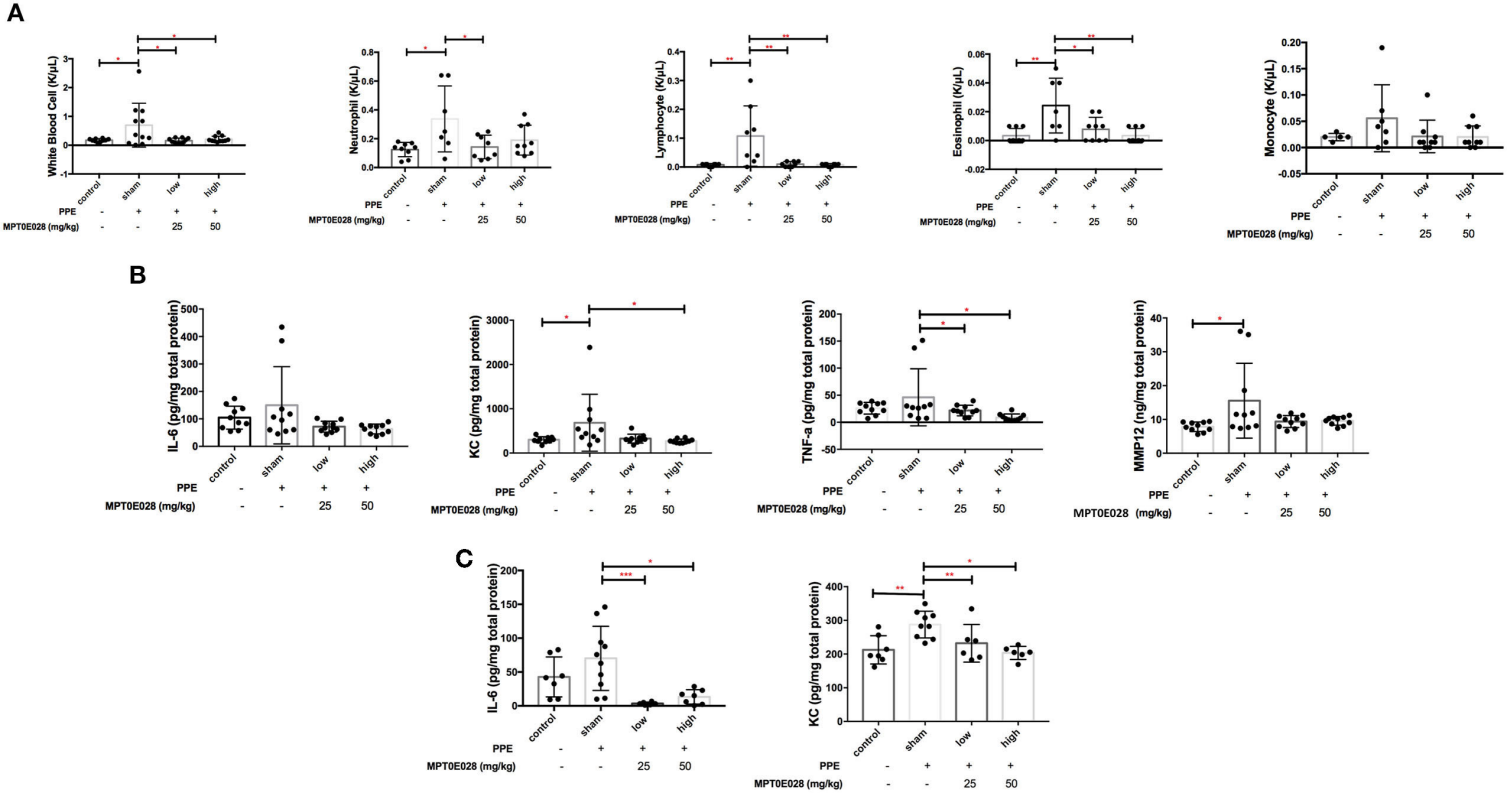
Inflammation by the MPT0E028 in mice with porcine pancreatic elastase (PPE)-induced emphysema. **(A)** White blood cells, neutrophils, lymphocytes, and eosinophils in bronchoalveolar lavage fluid (BALF) by the MPT0E028 at 0, 25, and 50 mg/kg body weight (BW) (*n* = 10). **(B)** Interleukin (IL)-6, keratinocyte-derived chemokine (KC), tumor necrosis factor (TNF)-α, and matrix metallopeptidase (MMP)-12 in lung lysates by the MPT0E028 at 0, 25, and 50 mg/kg BW (*n* = 10). **(C)** IL-6 and KC in serum by the MPT0E028 at 0, 25, and 50 mg/kg (*n* = 10). * *p* < 0.05; ** *p* < 0.01.

Figure 3A shows cell populations in the BALF of mice. Significant increases in white blood cells, neutrophils, eosinophils, and lymphocytes in BALF were observed in emphysematous mice compared to those in control animals (*p* < 0.05). We found that increasing levels of white blood cells, neutrophils, eosinophils, and lymphocytes in the BALF of emphysematous mice were significantly reduced by both 25 and 50 mg/kg BW of the MPT0E028 (*p* < 0.05).

### The MPT0E028 Decreased Inflammatory Responses

Changes in inflammatory cytokines and chemokines by the MPT0E028 in lung lysates and serum are presented in Figures 3B,C. We found that serum levels of KC and tissue levels of MMP-12 were significantly higher in emphysematous mice than in control animals (*p* < 0.05). Significant decreases in tissue levels of KC and TNF-α by 50 mg/kg BW of the MPT0E028 were observed (*p* < 0.05), and there were also significant reductions in serum levels of IL-6 and KC with the high-dose MPT0E028 treatment (*p* < 0.01). However, there was no significant difference in tissue levels of IL-6 between the groups.

### Regulation of Components of the Hippo Signaling Pathway in Lungs by the MPT0E028

**Figure 4:**
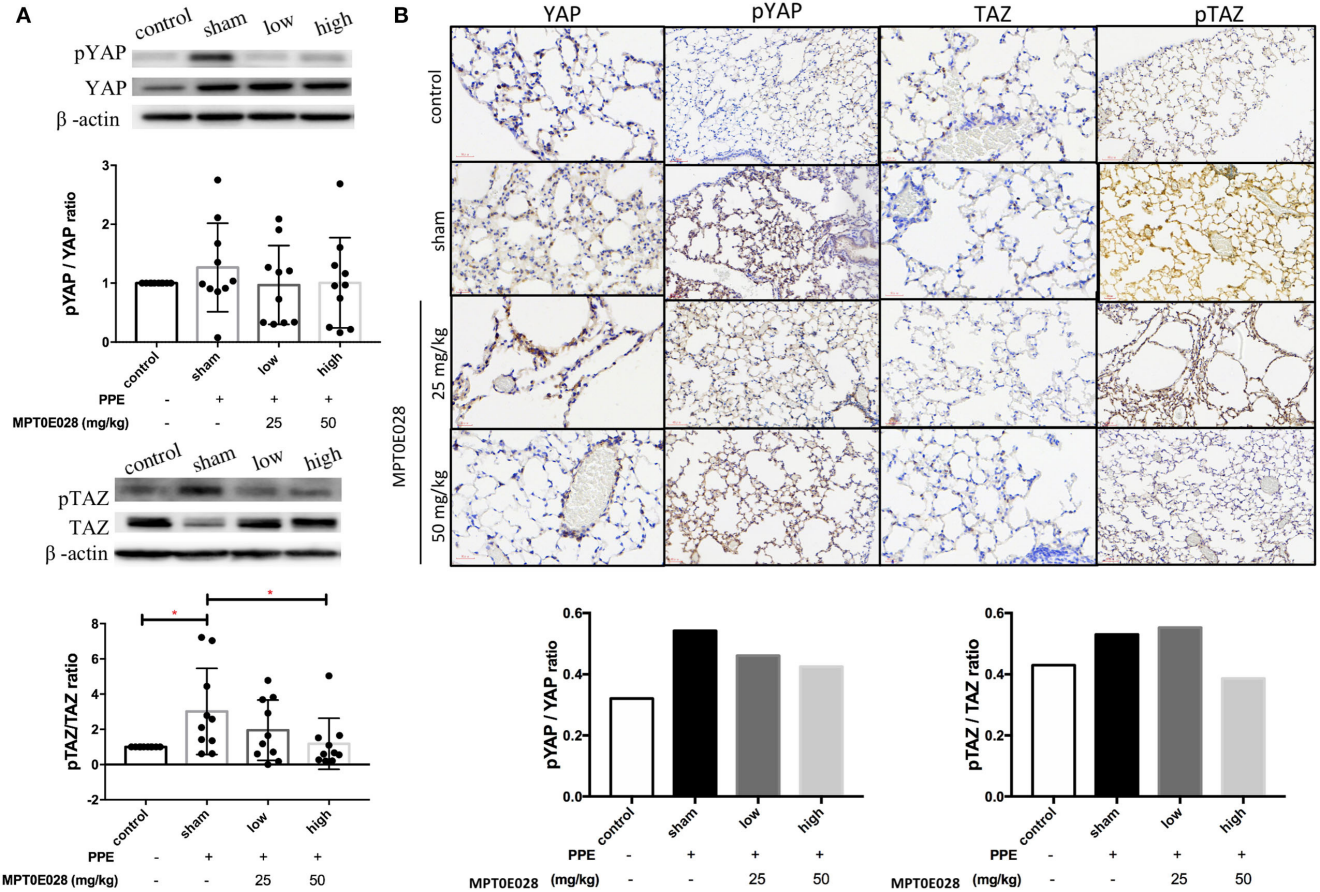
Expressions of phosphorylated (p)-yes-associated protein (YAP) and p-transcriptional coactivator with a PDZ-binding motif (TAZ) in the lungs and alveolar regions by the MPT0E028 in mice with porcine pancreatic elastase (PPE)-induced emphysema. **(A)** Expressions of p-YAP/YAP and p-TAZ/TAZ in whole lung lysates by the MPT0E028 at 0, 25, and 50 mg/kg body weight (BW) (*n* = 10). **(B)** Expressions of p-YAP/YAP and p-TAZ/TAZ in alveolar regions by IHC by the MPT0E028 at 0, 25, and 50 mg/kg BW (*n* = 1) (magnification X20). * *p* < 0.05; ** *p* < 0.01.

Figure 4 indicates that the MPT0E028 regulated expressions of components of the Hippo signaling pathway in the lungs. In Figure 4A, the value of p-TAZ/TAZ in whole lung tissue of emphysematous mice significantly increased compared to the control (*p* < 0.05). After treatment with the MPT0E028, we observed that the value of p-TAZ/TAZ with high-dose treatment (50 mg/kg BW) with the MPT0E028 had significantly decreased (*p* < 0.05). However, since the value of p-YAP/YAP and p-TAZ/TAZ in the alveolar region by IHC were calculated by the ratio of mean density for each slide, there was no significant differences in both of them.

### The MPT0E028 Activates SPC and T1-α Expressions in Lung Tissues

**Figure 5:**
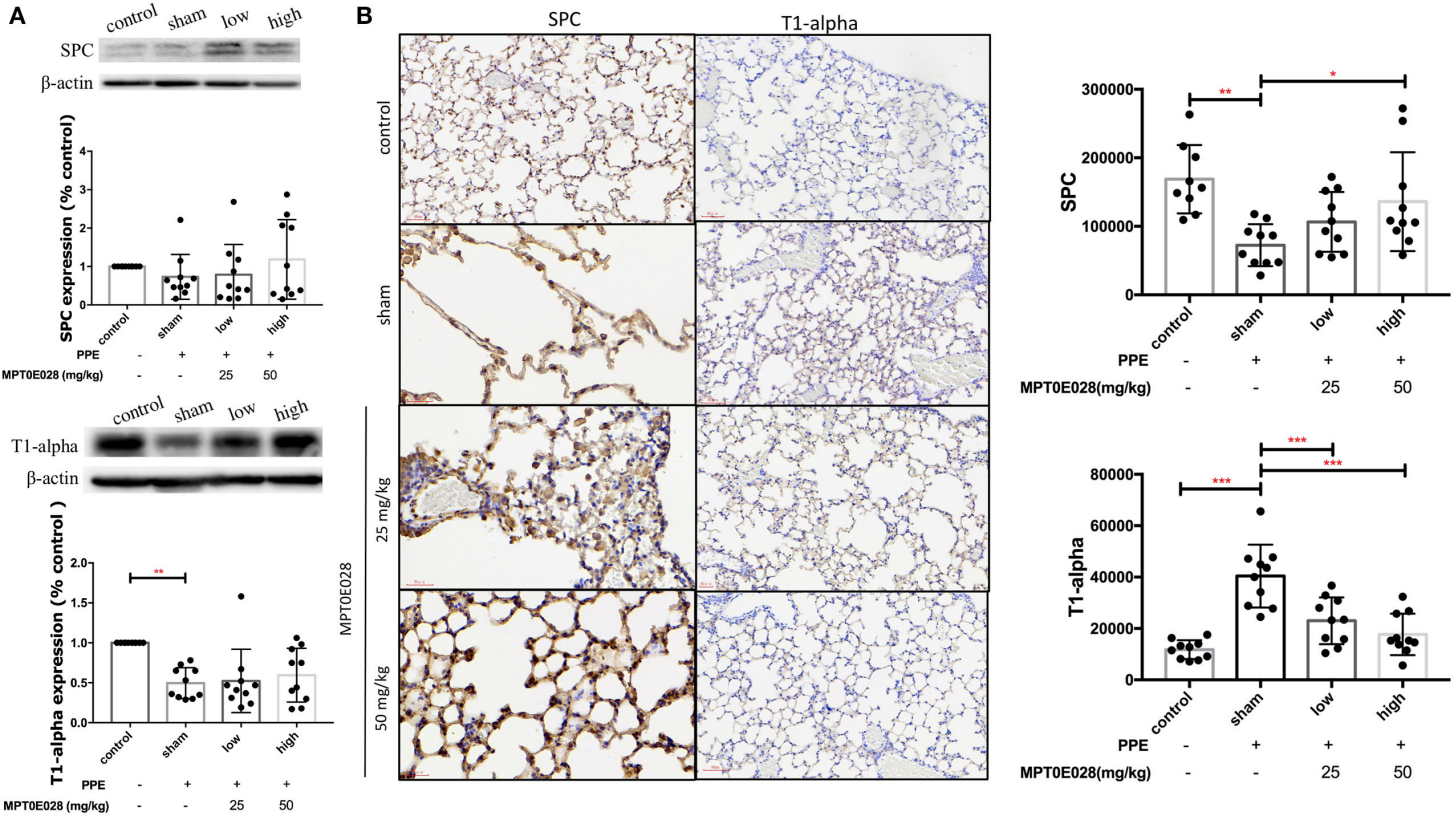
Expressions of surfactant protein C (SPC) and T1α in the lungs and alveolar region sby the MPT0E028 in mice with porcine pancreatic elastase (PPE)-induced emphysema. **(A)** Expressions of SPC and T1α in whole lung lysates by the MPT0E028 at 0, 25, and 50 mg/kg body weight (BW) (*n* = 10). **(B)** Expressions of SPC and T1α in alveolar regions by IHC by the MPT0E028 at 0, 25, and 50 mg/kg BW (*n* = 10) (magnification X20). * *p* < 0.05; ** *p* < 0.01; *** *p* < 0.001.

Figure 5 shows expressions of SPC and T1-α in lung tissues. As illustrated in Figure 5A, we observed no significant difference in SPC among the groups in whole lung tissues by Western blotting. However, SPC^+^ cells had significantly decreased in the alveolar region of emphysematous mice as shown in Figure 5B (*p* < 0.01). After being treated with 50 mg/kg BW of the MPT0E028, a significant increase in SPC^+^ cells was detected in the alveolar region (*p* < 0.05). Moreover, T1-α expression of whole lung tissues in Figure 5A by Western blotting was found to have decreased in emphysematous mice. However, there was no significant difference between groups after administrating the MPT0E028. When we focused on T1-α expression in the alveolar region (Figure 5B), a significant increase of T1-α^+^ cells by IHC was observed in emphysematous mice (*p* < 0.001), and expression of T1-alpha^+^ cells was significantly decreased by 25 and 50 mg/kg BW of the MPT0E028 in the alveolar region (*p* < 0.001).

### MPT0E028 Decreased p53 Expression in the Lungs

**Figure 6:**
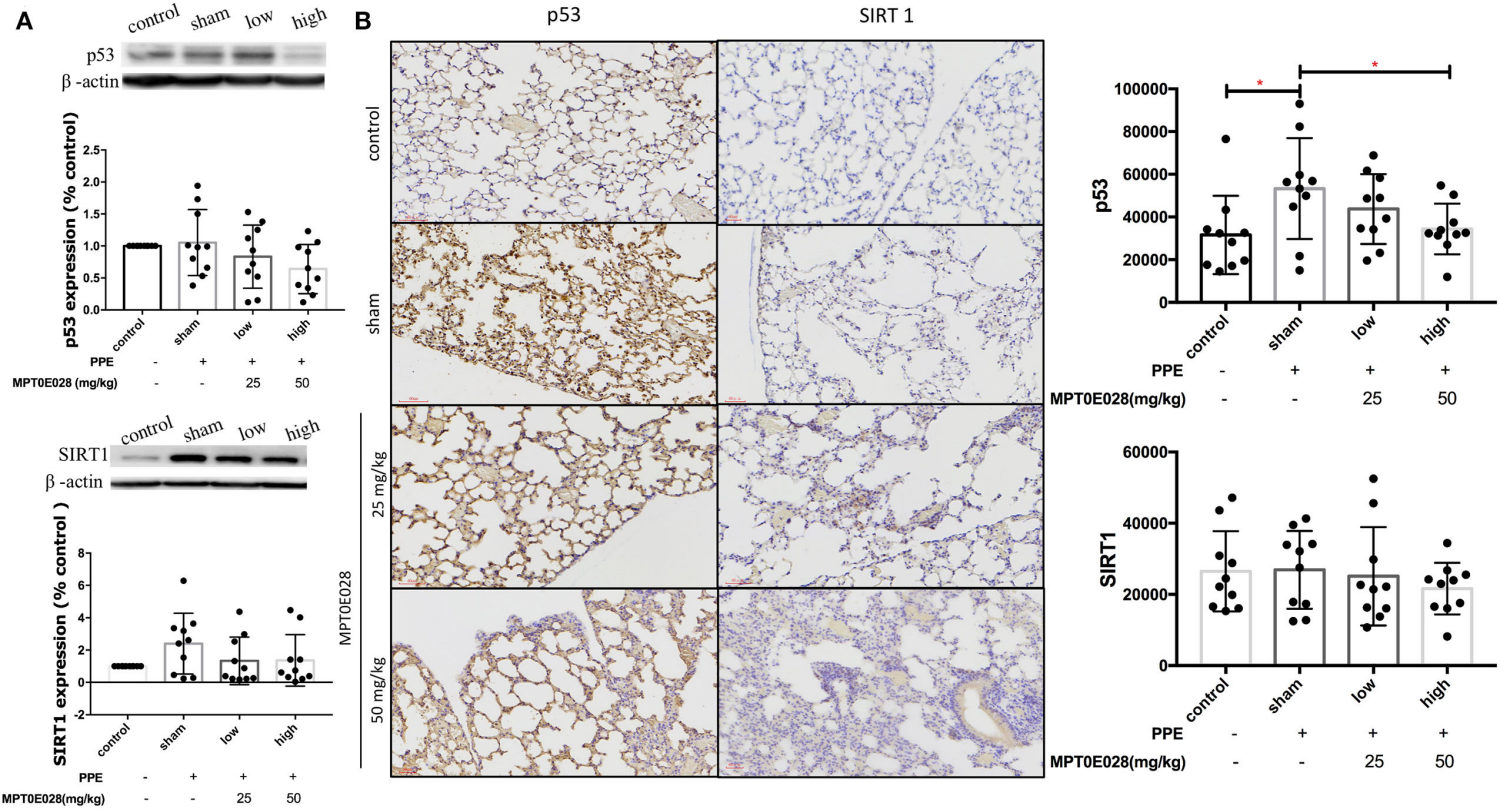
Expressions of sirtuin 1 (SIRT1) and p53 in the lungs and alveolar regions by the MPT0E028 in mice with porcine pancreatic elastase (PPE)-induced emphysema. **(A)** Expressions of SIRT1 and p53 in whole lung lysates by the MPT0E028 at 0, 25, and 50 mg/kg body weight (BW) (*n* = 10). **(B)** Expressions of SIRT1 and p53 in alveolar regions by IHC by the MPT0E028 at 0, 25, and 50 mg/kg BW (*n* = 10) (magnification X20). * *p* < 0.05.

Figure 6 demonstrates expressions of p53 and SIRT1 in the lungs. As illustrated in Figure 6A, there was no significant difference in SIRT1 expressions, or in the expression of p53 among the groups according to Western blotting results. Notably, we observed that the expression of p53^+^ cells by IHC had significantly increased in the alveolar region of mice with PPE-induced emphysema compared to the control (*p* < 0.05) (Figure 6B). After administrating the MPT0E028, we found that p53^+^ cells were significantly reduced by the high-dose treatment (50 mg/kg BW) of the MPT0E028 in the alveolar region (*p* < 0.05). However, there was no significant difference in SIRT1^+^ cells in the alveolar region.

## Discussion

Our findings revealed that the potent HDAC inhibitor MPT0E028 significantly increased the BW, reduced the emphysema severity, increased pulmonary function, and mitigated pulmonary inflammation in emphysematous mice. We also demonstrated for the first time that the MPT0E028 regulated components of the Hippo signaling pathway, which could be associated with a reduction in alveolar destruction. The results suggest that the MPT0E028 could have potential for reducing emphysema severity in COPD.

COPD is characterized as an inflammatory disease that involves airflow obstruction, which may lead to the use of accessory respiratory muscles with muscle wasting as well as BW loss. First, we observed that the MPT0E028 increased the BW and improved lung functions by reducing the tidal volume in emphysematous mice. Because of the imbalance between energy intake requirements and expenditures, 22~52% of COPD patients develop weight loss or have a normal weight with depletion of the fat-free mass (36). They may have difficulty in functions such as walking a distance and respiratory function, and also a reduced diaphragmatic mass. In addition, to prevent small airways from collapsing, COPD patients demonstrate pursed-lip breathing which increases the tidal volume associated with increasing the end inspiratory rib cage volume and reducing the end expiratory rib cage and abdominal volumes (37), or increasing the end inspiratory chest wall volume without changing the end expiratory chest wall volume (38). A previous study showed that gaining more than 2.0 kg improved respiratory muscle strength and quality of life among patients with COPD (39). An increased tidal volume may be found in COPD patients (40), which is also a sign of the development of the disease. Therefore, our findings indicate that the MPT0E028 could have the potential to reduce the functional decline of the lungs in emphysema patients.

There are two manifestations of COPD: chronic bronchitis and emphysema. Notably, we discovered that the MPT0E028 reduced the emphysema severity according to chest CT images and the MLI. Our results suggest that the MPT0E028 may be able to reduce emphysema severity *in vivo*. The reduction in alveolar destruction by the MPT0E028 may also result in improvements in lung function as results of the present study show. Previous *in vivo* studies reported that low-attenuation areas on quantitative CT images were correlated with relative volumes of emphysema and gas trapping (41, 42), especially with treatment of live emphysematous mice in longitudinal studies (43). In addition, since the MLI is a direct and unbiased estimation of a quantitative analysis of the lung structure, it was also measured in emphysematous mice in order to understand the distal air space size and alveolar density to support the above findings (44). Hence, decreasing emphysema's effect on the effective capacity might reveal that the MPT0E028 could reduce the severity of emphysema.

Neutrophilic inflammation is viewed as the initial destructor of the elastic matrix of alveoli (45). Also, eosinophil and lymphocyte counts were increased in induced sputum and peripheral blood in COPD patients (7). We observed decreased infiltration of neutrophils, lymphocytes and eosinophils by the MPT0E028 in emphysematous mice. The results suggested that the MPT0E028 was able to mitigate neutrophilic inflammation in emphysematous lungs. Therefore, we next examined the inflammatory response in the lungs and circulating system of mice. Notably, we found decreased inflammatory responses (i.e., KC, TNF-α, and MMP-12 in lung tissues and IL-6 and KC in serum) by the MPT0E028. Increasing levels of TNF-α worsen the severity of emphysema via recruiting inflammatory cells and then degrading more alveolar walls (46), especially during the period of exacerbation (47). Moreover, elevated IL-6 in the serum revealed a potential association with mortality due to COPD (48), and it was also inversely correlated with lung function and clinical outcomes in COPD patients (49, 50). Inflammatory inhibitors for leukotriene B4, TNF-α, IL-1, and IL-8 were shown to mitigate inflammation *in vivo* (51), and chronic treatment with inhaled corticosteroids also decreased the exacerbation (52). Consequently, the MPT0E028 could decrease inflammatory responses in emphysema.

We observed that the MPT0E028 regulated components of the Hippo signaling pathway in lung tissues of our emphysema model. YAP and TAZ are the main downstream mediators of the Hippo pathway and are responsible for the transition of alveolar epithelial type II to type I cells (53). They accumulate in nuclei, where they interact with transcription factors, such as TEA domain (TEAD) family members, and activate gene expressions to promote differentiation required for alveolar repair (54). Activating the YAP/TAZ signaling cascade can upregulate regeneration in response to lung injuries, such as inflammation (55, 56). YAP/TAZ might also play a role in an endogenous anti-inflammatory mechanism to limit the production of inflammatory mediators from AECIIs (15). Accordingly, the Hippo signaling pathway was regulated by the MPT0E028 in emphysema.

We next observed improvements in SPC and T1α expressions by the MPT0E028 in alveolar regions of emphysematous mice. SPC is secreted and mostly expressed by AECIIs, which are involved in alveolar epithelial regeneration after injury (57), to maintain the surfactant structure during the respiratory cycle (58). A previous study showed that SPC knock-out mice developed a severe progressive pulmonary disorder (59). This indicated that tissue levels of SPC might not only account for the number of AECIIs, but also be related to disease progression in COPD patients (60). T1α is mostly expressed by AECIs and inflammatory cells to upregulate leukocytes during inflammation in the lungs (61). It is also associated with detecting increased alveolar lymphatic vessels in COPD patients (62), which transport leukocytes to ectopic lymphoid aggregates in COPD lungs. A previous study showed that T1α-targeted therapies may have a beneficial effect of regulating the severity of lung inflammation (63). Hence, in our results, T1α expression in whole lung tissues by Western blotting might indicate an increasing number of AECIs; on the other hand, levels of T1α^+^ cells revealed decreasing severity of emphysema. As a consequence, the increasing levels of SPC and T1α by the MPT0E028 might show the potential effect on repairing alveolar epithelial cells in emphysema. However, more evidence is required to support this observation.

Senescence and apoptosis also play important roles in the development of emphysema. We found decreased expression of p53, but not SIRT1, by the MPT0E028 in alveolar regions of emphysematous mice. This observation suggests that the MPT0E028 may mitigate apoptosis. SIRT1 stimulated p53 deacetylation, and acetylation of p53 enhanced cell cycle arrest, cellular senescence, and apoptosis (64). In COPD patients, it was found that p53 increased in the ACEIIs of COPD patients (65), and alveolar apoptosis increased despite smoking cessation (66). Hence, antagonism of p53-mediated apoptosis would be a promising therapeutic intervention (67, 68). Together, the MPT0E028 reduced p53 activation in lungs of emphysematous mice, which may be associated with mitigating alveolar destruction in those mice.

There are some limitations in our study. Inhibition of class 1 and 2 HDAC was also shown to cause emphysema in rats (69), further studies should be investigated to clarify the effect of different dosage of MPT0E028. In this study, we analyzed whole-lung lysates and quantified IHC expression to examine protein expressions in the lungs and alveolar regions. *In vitro* work should be conducted in the future to confirm the observations in the present study. Relationships of the Hippo signaling pathway with pathways for alveolar repair, proliferation, and apoptosis should be examined in the future. The effects of the MPT0E028 on COPD patients with emphysema are unclear and should be investigated in the future.

## Conclusions

In conclusion, our results suggest that the MPT0E028 reduced emphysema severity with improvements in lung function and weight loss as well as reduced inflammation in mice. Increasing YAP/TAZ and SPC expression, and decreasing p53 expression were also observed in emphysematous mice. The potent MPT0E028 may have therapeutic potential in emphysema.

## Data Availability Statement

The raw data supporting the conclusions of this article will be made available by the authors, without undue reservation.

## Ethics Statement

The animal study was reviewed and approved by Institutional Animal Care and Use Committee of Taipei Medical University.

## Author Contributions

L-YY and H-CC contributed to the completion of interpretation of the data and the manuscript. H-CC, C-HLin, and KC contributed substantially to the concept, design, interpretation of the data, and completion of the study and manuscript. L-YY, H-SL, and C-HLiu contributed to the animal experiments. Y-TF, Y-YC, and Y-CL contributed to the chest image analyses. VL contributed to the histological analyses. J-PL contributed to the chemical preparation. All authors contributed to the critical revising of the manuscript for important intellectual content and have read and approved the final manuscript.

## Funding

This study was funded by the Ministry of Science and Technology of Taiwan (109-2314-B-038-093-MY3).

## Conflict of Interest

The authors declare that the research was conducted in the absence of any commercial or financial relationships that could be construed as a potential conflict of interest.

## Publisher's Note

All claims expressed in this article are solely those of the authors and do not necessarily represent those of their affiliated organizations, or those of the publisher, the editors and the reviewers. Any product that may be evaluated in this article, or claim that may be made by its manufacturer, is not guaranteed or endorsed by the publisher.
