# Supplementary material for: A Potent Histone Deacetylase Inhibitor MPT0E028 Mitigates Emphysema Severity via Components of the Hippo Signaling Pathway in an Emphysematous Mouse Model
Source: Front Med (Lausanne). 2022 May 18;9:794025. doi: 10.3389/fmed.2022.794025 (PMC9157428; doi:10.3389/fmed.2022.794025)
Supplement: Supplementary file 1 [file Data_Sheet_1.docx]

**A potent histone deacetylase inhibitor MPT0E028 mitigates emphysema severity via components of the Hippo signaling pathway in an emphysematous mouse model**

Lu-Yang Yeh^1^, Yu-Ting Fang^2^, Hong-Sheng Lee^3^, Chia-Hao Liu^3^, You-Yin Chen^2^, Yu-Chun Lo^4^, Vincent Laiman^5,6^, Jing-Ping Liou^7^, Kian Fan Chung^8^, Hsiao-Chi Chuang^9,10,11*^, Chien-Huang Lin^3^

^1^School of Medicine, College of Medicine, Taipei Medical University, Taipei, Taiwan

^2^Department of Biomedical Engineering, National Yang Ming Chiao Tung University, Taipei, Taiwan

^3^Graduate Institute of Medical Science, College of Medicine, Taipei Medical University, Taipei, Taiwan

^4^PhD Program for Neural Regenerative Medicine, College of Medical Science and Technology, Taipei Medical University, Taipei, Taiwan.

^5^International PhD Program in Medicine, College of Medicine, Taipei Medical University, Taipei, Taiwan

^6^Department of Anatomical Pathology, Faculty of Medicine, Public Health, and Nursing, Universitas Gadjah Mada, Yogyakarta, Indonesia

^7^School of Pharmacy, College of Pharmacy, Taipei Medical University, Taipei, Taiwan

^8^National Heart and Lung Institute, Imperial College London, London, UK

^9^School of Respiratory Therapy, College of Medicine, Taipei Medical University, Taipei, Taiwan

^10^Division of Pulmonary Medicine, Department of Internal Medicine, Shuang Ho Hospital, Taipei Medical University, New Taipei City, Taiwan

^11^Cell Physiology and Molecular Image Research Center, Wan Fang Hospital, Taipei Medical University, Taipei, Taiwan

**Running Head:** Alveolar repair by MPT0E028 in COPD

**Word count:** 4,058 [excluding abstract (242 words) and references]; 6 figures.

***Corresponding Authors**

*Hsiao-Chi Chuang, PhD*

Inhalation Toxicology Research Lab (ITRL), School of Respiratory Therapy, College of Medicine, Taipei Medical University, 250 Wuxing Street, Taipei 11031, Taiwan.

Telephone: +886-2-27361661 ext. 3512. Fax: +886-2-27391143. E-mail: [chuanghc@tmu.edu.tw](mailto:chuanghc@tmu.edu.tw)


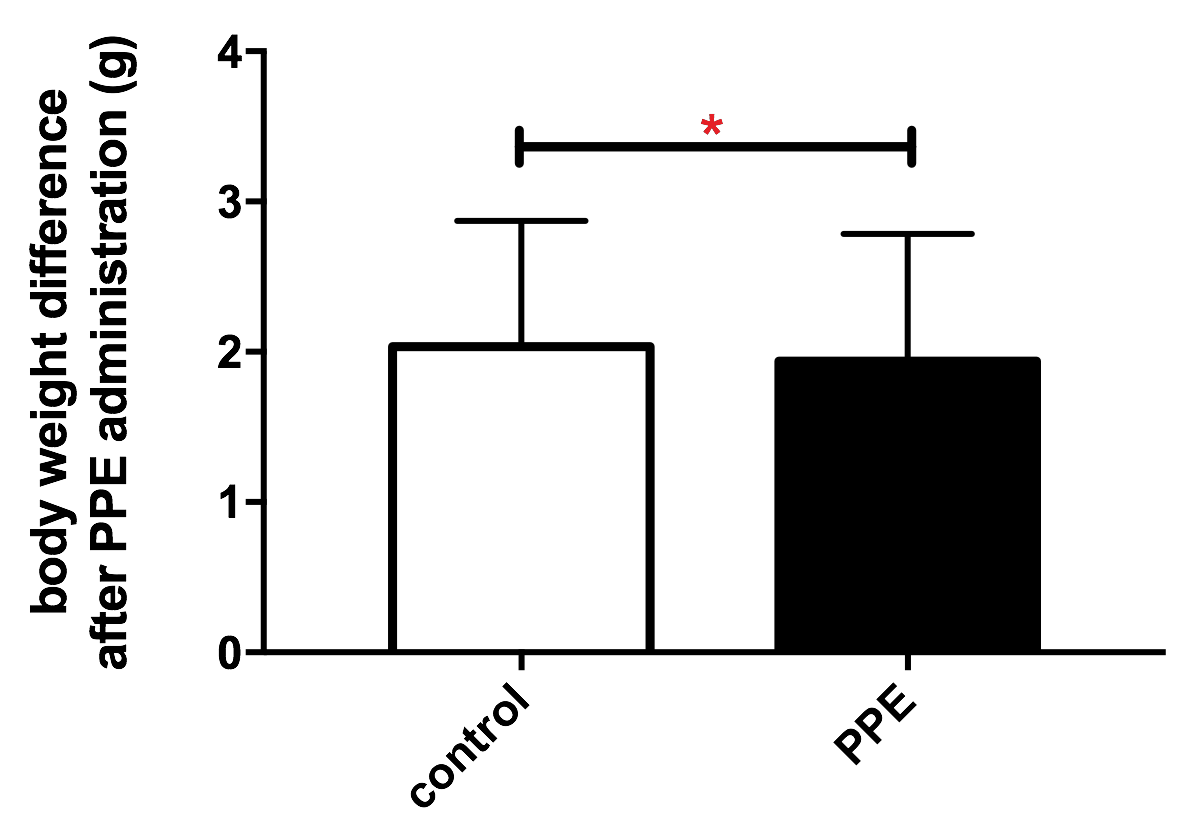


**Figure S1.** **Body weigh difference between control and PPE groups.**

Body weight (BW) differences after administration with PPE.* *p*<0.05
